# A Case Report Describing Three Cases of Challenging or Failed Intubation after Cervical Spine Surgery: A Peril of Early Extubation

**DOI:** 10.1055/s-0039-1700806

**Published:** 2019-11-13

**Authors:** Puneet Mishra, Kelly Louise Mishra, Cassandra Palmer, Amy Robertson

**Affiliations:** 1 Department of Anesthesiology, Vanderbilt University Medical Center, Nashville, Tennessee

**Keywords:** cervical spine surgery, reintubation, airway complications

## Abstract

Postoperative airway complications can be a common, yet perhaps underappreciated, complication in patients undergoing cervical spine surgery. Presented here are three cases in which patients experienced postoperative airway compromise, resulting in difficulty establishing a secure airway following cervical spine operations. Establishing factors that contribute to airway complications after cervical spine surgery can aid in early identification of high-risk patients to create an appropriate airway management strategy. Ultimately, the frequency of airway difficulty after removal of the endotracheal tube in patients undergoing cervical spine surgery should not be taken lightly.

Cervical spine surgery can lead to airway obstruction, which is often secondary to hematoma or oropharyngeal and laryngeal swelling. 1 2 Although more likely to occur with a combined anterior and posterior discectomy and fusion, obstruction can still manifest after either an anterior or posterior approach. When airway obstruction occurs, it is most common in the first 6 hours but may present even later. 3 One chart review demonstrated a 6% incidence of the airway complications in anterior cervical spine surgery, with one-third of these cases requiring reintubation. 4 Another chart review showed that 1.75% of the patients needed prolonged intubation and 1% required reintubation postoperatively. 5 Although the reported incidence of airway compromise is low, the clinical significance of outcomes may be catastrophic. In addition to potentially difficult or failed urgent reintubation, adverse outcomes may include prolonged hypoxia, pneumonia, tracheostomy, longer intensive care unit stay, and death. 6 7 Thus, identifying postoperative patients who are at risk for potential airway complications is an important clinical determination. Furthermore, establishing a specific airway management plan may lead to improved outcomes.

## Case Description

### Case 1

A 71-year-old male with a past medical history of anterior cervical fusion presented after a trauma for an occipital to C4 posterior spinal fusion. He had a body mass index (BMI) of 30, had an American Society of Anesthesiology (ASA) classification of 4, was a nonsmoker, and had a history of coronary artery disease, hypertension, and chronic pain. Preoperative intubation was easy using a video laryngoscope with a Cormack–Lehane grade I indirect view.

After an uneventful operation, the patient returned to the intensive care unit (ICU) intubated. Five hours later, he was felt to meet extubation criteria. However, before the endotracheal tube was electively removed, he became agitated and pulled the endotracheal tube out of his airway himself. Initially, he was alert with adequate oxygenation and ventilation. Therefore, the ICU team elected not to immediately reintubate. Ninety minutes later, urgent reintubation was required for hypoxic respiratory failure.

Intubation with a video laryngoscope proved impossible, and it was noted that the pharynx appeared very edematous. Adequate ventilation and oxygenation were achieved with a rescue supraglottic airway. Fiberoptic intubation was attempted and significant airway edema was observed and intubation was not successful. The trauma surgery team was called, which performed an emergency tracheostomy at bedside.

### Case 2

A 54-year-old female with a C5-C6 cervical disc herniation presented for anterior spinal fusion. She had a BMI of 39, had an ASA classification of 3, a Mallampati score of 4, was a smoker, and had a history of hypertension, transient ischemic attack, and diabetes. Despite a history of difficult intubation, she was intubated in the first attempt with a video laryngoscope. She was extubated awake and transported to the postanesthesia care unit.

On postoperative day 1, the patient developed a neck hematoma, began experiencing dyspnea and stridor, and was taken to the operating room. No identifiable airway structures were visualized on intubation attempt using a video laryngoscope. Mask ventilation was unsuccessful. Surgeons opened the neck incision to decompress the hematoma and the patient was able to be ventilated with a supraglottic airway. Fiberoptic intubation was successful. A significant amount of soft tissue and airway edema was noted.

### Case 3

A 55-year-old male presented with an atlanto-occipital dissociation. He had a BMI of 29, had an ASA classification of 3, a Mallampati score of 3, was a nonsmoker, and had a history of emphysema. He had been intubated by paramedics in the field. He was brought to the operating room for an occiput-to-C2 posterior arthrodesis. The operative course was uneventful. Postoperatively, he was transferred to the ICU intubated, and he was extubated the next day. Twelve hours later his oxygen saturation and mental status declined prompting the team to attempt reintubation. Two intubation attempts with a video laryngoscope were unsuccessful and mask ventilation was challenging. The team proceeded with an emergent cricothyroidotomy, and the patient was then taken to the operating room for a tracheostomy.

## Discussion

### Why Is Airway Compromise a Problem after Cervical Spine Surgery?

Since cervical spine surgery occurs in the same anatomical region as the larynx, and often patients are in the prone position with associated edema, there is a risk for the development of postoperative airway compromise. During an anterior approach for cervical spine surgery, access to the vertebral column is often achieved with medial retraction of the upper airway structures and esophagus. 2 Potential complications associated with this approach include: airway edema, recurrent laryngeal nerve palsy, dysphonia, dysphagia, esophageal perforation, hoarseness, and sore throat. 8

Angioedema is the earliest cause of airway impairment postoperatively, occurring in 6 to 12 hours. Between 6 and 24-hours postoperatively, patients are at risk for retropharyngeal hematoma. Pharyngolaryngeal edema is the most likely cause of airway compromise in the 24 to 72-hour period. 9 Clinical manifestations of upper airway edema vary. Early signs typically include dyspnea and difficulty speaking. As airway edema progresses: hypoxia, hypercarbia, inspiratory stridor, and cyanosis can develop. Restlessness and agitation can develop secondary to hypercarbia. Without immediate attention and proper intervention, respiratory failure and arrest can occur. 3 The typical time course for the occurrence of upper airway edema is 12 to 72 hours. 2

As demonstrated in these cases, edema can compromise the respiratory status of patients and distort airway anatomy such that establishing a secure airway becomes exponentially more challenging. If a patient develops respiratory compromise, the initial method of intubation may no longer be successful due to either difficulty visualizing anatomical landmarks, or the inability to pass the endotracheal tube into the trachea. Therefore, anesthesiologists and surgeons should have a high degree of vigilance regarding the risks of early extubation after cervical spine surgery.

### What Are Specific Predictors for Postoperative Airway Complications after Cervical Spine Surgery?

Review of the medical literature reveals various predictors of postoperative airway complications. Risk factors that are predictive for postoperative airway compromise after cervical spine surgery include: (1) location of surgery, (2) duration of surgery (over 10 hours), (3) intraoperative blood loss, (4) rheumatoid arthritis, (5) obesity, (6) asthma, (7) patient age, (8) blood transfusion, (9) neurological abnormality preoperatively, (10) male gender, (11) dependent functional status, (12) chronic obstructive pulmonary disease (COPD), (13) bleeding disorder, and (14) ASA classification> 2. 10 11 12

### What Are Safe Extubation Practices in High-Risk Patients?

An “at risk” extubation is a situation in which the ability of the patient to maintain airway patency or oxygenation is uncertain. 13 Risk factors include preexisting airway difficulties, perioperative airway deterioration, and restricted airway access. 4 Strategies for airway management after cervical spine surgery are summarized in Table 1 .

**Table 1:** Airway management strategies after cervical spine surgery

#	Strategy
1	Assess for risk factors of increased likelihood of airway complications.
2	Perform cuff leak and/or fiberoptic visualization to assess for pharyngeal or tracheal swelling.
3	Delay extubating if there is any clinical suspicion of airway edema.
4	Consider administration of steroids if significant edema is present.
5	Have a low threshold for early reintubation if patient demonstrates signs/symptoms of respiratory compromise.
6	Prior to extubation, establish an airway management plan if the patient requires reintubation.
7	Consider performing extubation over an airway exchange catheter to facilitate reintubation and oxygenation.
8	Have advanced airway devices immediately available.
9	Notify surgical airway team early if necessary.

A cautious approach of leaving high-risk patients intubated for close airway monitoring until airway swelling subsides may be optimal. 11 Kim et al recommended that a prolonged intubation for high risk patients even without respiratory compromise is the safest practice. 5 Suk et al evaluated 87 patients with serial X-rays after undergoing anterior cervical discectomy and fusion (ACDF) and found that prevertebral soft tissue swelling increased markedly on the second postoperative day and peaked on postoperative days 2 and 3. 14 Prior to extubation, an endotracheal tube cuff leak test may be helpful in identifying patients with significant airway edema. A cuff test compares expiratory tidal volumes with the cuff inflated to the tidal volumes with the cuff deflated, looking for a reduction of 15% in tidal volume, to indicate an appropriate leak is present when the cuff is deflated. 15

The beneficial effect of steroids on reducing airway edema after spine surgery remains controversial. Nam et al compared the effect of postoperative dexamethasone on prevertebral soft tissue swelling with that of placebo in patients undergoing ACDF and demonstrated that steroids were not effective in reducing postoperative prevertebral soft tissue density but did reduce dyspnea in the immediate postoperative period. 16 Another study assessed postoperative airway edema with fiberoptic bronchoscopy after perioperative methylprednisolone administration. There was less severe pharyngeal edema in the methylprednisolone group. 17 Therefore, the clinical benefit of decreased airway complications associated with the routine administration of steroids remains unclear.

## Conclusion

These cases illustrate the significant complications that can arise from premature extubation after cervical spine surgery. A more cautious and prudent approach to extubating a patient after cervical spine surgery is recommended. Schroeder et al concluded that if a secure airway is restored quickly, the patients’ outcomes are mostly favorable. 18 Prior to extubation, the medical team should have an airway management plan in place in the event airway compromise occurs and reintubation is necessary. This plan should include having advanced airway devices immediately available, staff trained to identify at risk patients, and access to a surgeon capable of performing a surgical airway.
